# A novel flax fibre composite material for stringed instrument fingerboards

**DOI:** 10.1177/07316844211067540

**Published:** 2022-03-04

**Authors:** Olivier Chabot, Larry Lessard, Martine Dubé

**Affiliations:** 1 CREPEC, Department of Mechanical Engineering, 14849 École de Technologie Supérieure, Montréal, QC, Canada; 2 CREPEC, Department of Mechanical Engineering, 5620 McGill University, Montréal, QC, Canada

**Keywords:** Flax fibre, biocomposite, stringed instrument

## Abstract

With growing restrictions on the exploitation and trade of current stringed musical instruments fingerboard materials such as ebony and rosewood, for economical and ethical reasons, instrument makers are looking for alternative materials. The present work describes the development of a homogeneous 60% bio-based flax fibre composite material with physical properties similar to commonly used fingerboard woods. As a proof of concept, prototype guitar neck was built using the material, demonstrating its compatibility with existing guitar manufacturing techniques.

## Introduction

The current state of stringed musical instrument designs has little evolved in the past century, as they have already converged to the instruments used today, including the materials used.^1^ The usual guitar design includes various essences of wood, among them ebony *Dyospyros celebica* DYOSPYROS CELEBICA and rosewood *Dalbergia latifolia* DALBERGIA LATIFOLIA which are slow growing dense trees found in tropical regions, notably in Brazil and Madagascar.^2^

**Figure 1.**
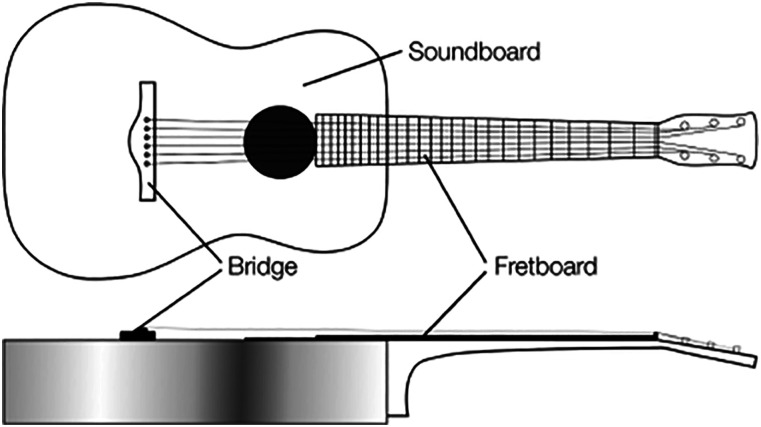
Key parts of the acoustic guitar.

These two woods are used primarily in the fingerboard (or fretboard) and the bridge due to their hardness and stiffness (see Figure 1). These parts are under considerable stress: the fingerboard is abraded by the strings during play and stiffens the instrument neck to withstand the string tension and the bridge is a key part fixing the strings onto the soundboard.

It is worth noting that the word fingerboard designates the part on which the string are pushed against for any type of stringed instrument, whereas the word fretboard designates the fingerboard of fretted instruments, such as the guitar. In this research, the word fingerboard will be used when referring to the general part of a stringed musical instrument and the word fretboard will be used to designate the specific guitar part.

The growing number of CITES (Convention on International Trade in Endangered Species) regulations on the commerce of exotic woods^3^ have made the supplying of exotic tone woods challenging for guitar makers who may fall back on unregulated sources, facing legal consequences.^4^ Growing regulations have also increased customer awareness.^5^ Linked issues such as the increase in illegal logging, endangering ecosystems in developing countries, especially those inhabited by aboriginal populations,^6^ have motivated stringed instrument makers to look for a synthetic alternative to conventional fingerboard woods.

Commercially available alternatives to wood for fingerboards already exist for guitar fretboards, where the most widely used is *Richlite*, a material made of paper infused with phenolic resin.^7^ The similarly named *Rocklite* is made of “wood fibres and thermoset resin”.^5^ According to guitar makers, the latter is more similar to wood than *Richlite* and easier to work with.^8^ The Corène material is similar to Richlite, but sold in shapes to accommodate classical stringed instruments makers, such as those building cellos and violins.^9^

According to Godin Guitars,^10^ Richlite is harder to work with than wood, both during shaping and gluing of the fretboard. As for Rocklite, it is used by artisan guitar makers, but it remains more expensive than exotic woods and, as of now it is not available on a commercial scale. Corène fingerboards present the same issues as Richlite and its synthetic nature makes it less popular to traditional instrument purchasers, who would rather use natural or bio-based materials. Thus, motivation remains to develop a bio-based material producible on an industrial scale and compatible with existing instrument fingerboard manufacturing processes.

The use of composite materials in musical instruments is well established, with commercial composite instruments dating back to Ovation guitars in 1966, with its glass fibre guitar body design.^11^ With wood being a natural composite material having orthotropic mechanical properties, it makes sense to try and replace it with synthetic composite materials which can be designed to have similar properties. Nowadays, the use of synthetic fibre composite materials in stringed instruments, especially guitars is widespread with numerous companies currently offering carbon fibre instruments, like Rainsong and KLOS,^12,13^ with prices in the same range as mid-scale wooden guitars. According to both company websites and KLOS founder Adam Klosowiak, the main sale points of carbon fibre composite acoustic guitars are their resistance to humidity and thermal stress, their loudness and their stability trough time. However, the low variability in the properties of carbon fibre composite materials can make the instrument seem ‘soul-less’ (a property that is difficult to measure) to customers used to wood and its natural variability.^14^ In addition, wood instruments are known to change in tonality over time as the wood creeps and follows cycles of moisture absorption,^15^ a phenomenon unseen in carbon fibre instruments.

Natural fibre composite materials are an excellent alternative to the traditional woods because they can be manufactured with the same techniques used for carbon fibre composite materials, but behave more like wood when subjected to environmental stress^16,17^ In a previous study, Philips^18^ demonstrated the suitability of flax fibre composite material as a stringed instrument material by developing an efficient manufacturing method for a soprano ukulele. With the existing motivations in replacing rosewood and ebony with cheaper, more ethical and readily available materials, subsequent research was focused on fingerboard material alternatives. Duraisamy^17^ and Ohlmann^19^ have done iterations on the design of a flax based composite material for stringed instrument fingerboards. Duraisamy^17^ studied the importance of the different material properties on the acoustic behaviour of the composite, as well as the environmental effects on prospective and actual fingerboard materials. Ohlmann^19^ provided hybrid flax-carbon-balsa composite material layups matching closely the acoustic behaviour of rosewood used for fretboard. However, according to head of design at Godin guitars Daniel Fiocco,^10^ because the fretboard is not a component of the guitar body and is not resonating with a high amplitude, its acoustic properties are less important than its physical properties.

Thus, the objectives of this research are to develop a synthetic alternative to the exotic woods used in the fingerboards of stringed instruments and to demonstrate its compatibility with existing instrument manufacturing techniques and processes.

## Developing a fingerboard material

In stringed instruments, the fingerboard serves two main purposes: Stiffen the instrument neck, which is normally manufactured using softer woods^20^ and provide a surface against which the musician can press the strings to play the instrument. The fingerboard material must be resistant to abrasion because the instrument strings are normally made of hard materials such as nylon and steel.^1^ For the material to be manufactured into a fretboard, it should be compatible with metal fret inserts which need to be pressed and glued into slots cut in the material. Fretboards make the instrument easier to play because they help the musician to always press the strings at the right length, as it is the case with the guitar.

The fingerboard surface is normally shaped into a compound radius shape by the luthier, then metal frets can be inserted and finally the surface is finished and polished to help the musician’s hand glide when playing and for aesthetics reasons.

### Requirements for a fingerboard material

A viable fingerboard material should be homogeneous trough the thickness so that it can be cut into radius shapes. Also, throughout the life of the instrument, it is common for a musician to have his fretboard sanded and refinished by a luthier.^8^ To ensure compatibility with existing infrastructures and traditional instrument crafting techniques, the material should be machinable with wood cutting tools, while still being hard enough to withstand the abrasion caused by prolonged playing by the musician. As the fingerboard is an important structural component of the instrument, it should be stiff enough to withstand the string tension, thus preventing creep in the long run which can deteriorate the sound quality of the instrument,^21^ as stringed instruments are meant to last decades. For the material to be used in the manufacturing of guitars or other fretted stringed musical instrument, it should be compatible with fret inserts, such as a thin slot can be easily machined trough the thickness and a metal fret can be pressed into the slot and bonded with polyvinyl adhesive.

As the visual aspect of musical instruments can impact the sound perception of players and listeners,^22^ aesthetics are important in guitar making. A material having a similar grain pattern and texture to wood is preferable to a completely homogeneous material, as it creates a perceived uniqueness associated with higher end instruments and validates the customer expectations.^8^ The material colour should match the expected dark wood colour associated to exotic woods such as rosewood and ebony, control on the material colour is desirable as it allows the material to be matched to different wood essences. According to Liu et al.,^23^ in their review of woods for stringed instrument fingerboards, the material surface colour should be “black, dark brown or dark purple-brown”, in order to be in accordance with traditional aesthetics.

### On the use of natural fibre reinforcement materials

As stated in literature review, works of Duraisamy and Ohlmann^17,19^ focussed on the use of flax fibre composite materials to replace Rosewood in musical instrument fingerboards. Flax fibre are widely available natural fibres, they are less dense than synthetic fibres and have a higher damping ratio, thus making them similar to wood. The current design of the fingerboard material was focused on flax fibre composite because its monolithic properties are similar to those of ebony and its fibres can be moulded to produce a material with wood-like texture.^24^ Moreover, the production of flax composite material reinforcements produces much less CO_2_ than synthetic fibres.^25,26^In fact, the CO_2_ produced during the manufacturing of flax fibre reinforcements can be offset by the CO_2_ absorbed during the growth of the flax plants.^27^

### Desired physical properties

To ensure the material is sufficiently stiff to maintain the structural integrity of the guitar, its mechanical properties should be close to those of ebony and rosewood.^19^ According to Liu et al.,^23^ the fingerboard material should have a density higher than 0.8 g/cm^3^, as density is correlated with wear resistance and hardness for woods. Considering this requirement, a synthetic fingerboard should have hardness and wear resistance equal to or higher than those of commonly used woods. Liu et al.^23^ have also addressed requirements on the hygroscopic behaviour of a fingerboard material, as the dimensional stability of parts used in a musical instrument is of great importance because a small change in dimension can affect the justness, play-ability and timbre of the instrument,^21^ the synthetic material should have hygroscopic properties similar to those of commonly used wood or be more stable to humidity, while still being less inert than carbon fibre composite materials, as to allow the material to have slight changes in mechanical behaviour while maintaining the structural integrity of the instrument. According to the study performed by Duraisamy,^17^ flax composite materials absorb significantly less moisture than Rosewood, a common fretboard material, and their resonant frequency varies in similar way, showing that the hygroscopic effect on their mechanical properties is similar.

## Flax fibre composite material

With all these requirements in consideration, a flax based composite material was designed. We used the Sicomin *Infugreen810* resin to obtain a larger bio-based fraction.^28^ The design iterations on the composite material were all performed using the vacuum assisted resin transfer moulding process (VARTM), as it allows the production of low porosity parts and is easily scalable for large production of planar parts, such as guitar fretboard blanks.

### Manufacturing

One of the principal challenges in manufacturing a flax composite fretboard material was the homogeneity trough thickness, as composite materials are usually laminated from a number of plies. Reaching the required part thickness from the fretboard blank measurements (533 mm x 73 mm x 9.9 mm) prescribed by Godin Guitars was also a challenge.

**Figure 2.**
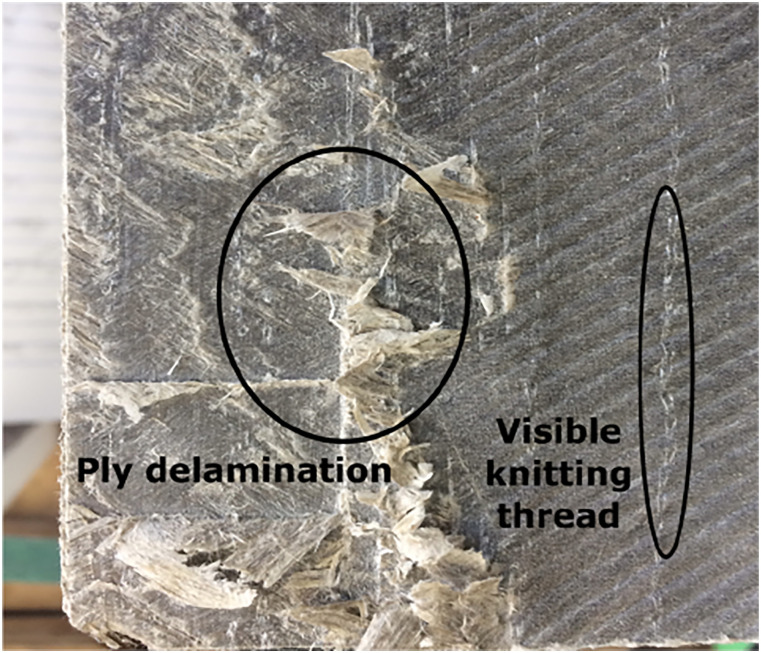
Delamination and visible non-crimp fabric stabilizing threads on a flax composite material sample.

The previous work of Ohlmann^19^ recommended the use of a [±25_*n*_] layup of flax composite material as a wood replacement for guitar fingerboards, a sample of flax composite material made from non-crimp fabric with a [±25_6_] layup was manufactured. The material was deemed not viable, because the differences in ply angles trough the thickness caused delamination when machining the part with common lutherie tools (wood planer and chisel), because low shear strength planes occurred between plies. Additionally, the knitting threads used to keep the flax fibres together in the fabric before infusion were visible in the manufactured part, which made it not suitable for a finished musical instrument (see Figure 2).

**Figure 3.**
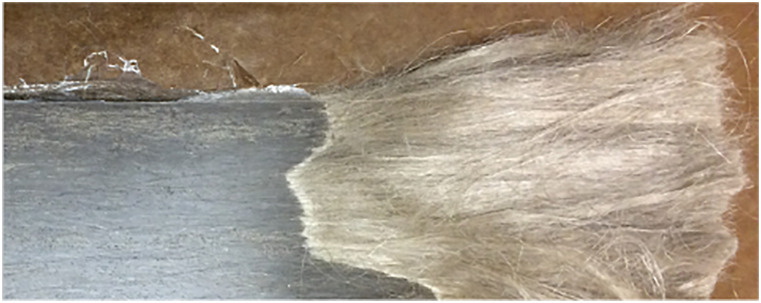
Sample made from oriented loose flax fibres (unfinished infusion to show wet and dry fibres).

Subsequently, another flax composite material was produced using oriented unbound flax fibres to reproduce the distribution of wood fibres in wood (see Figure 3). As the fibres were bundled into a single pack, there was no interface between plies, no knitting threads and the resulting material was homogeneous trough-the-thickness.

The loose fibres were obtained by removing the stabilizing threads from flax non-crimp composite reinforcement fabric. The resulting fibres were bundled to form an oriented composite part, as the fibres where stacked to ± 5 degrees locally, the wavy finish of natural wood fibres was reproduced. To obtain a planar part, a steel caul plate was used, the fibre bundle was placed on a planar mould and a plate with the same dimensions as the desired part was placed on the bundle. For this material, the vacuum assisted infusion was performed without infusion media, as the permeability across the part in the fibre direction was sufficient to ensure infusing the whole part during the resin work time.

**Figure 4.**
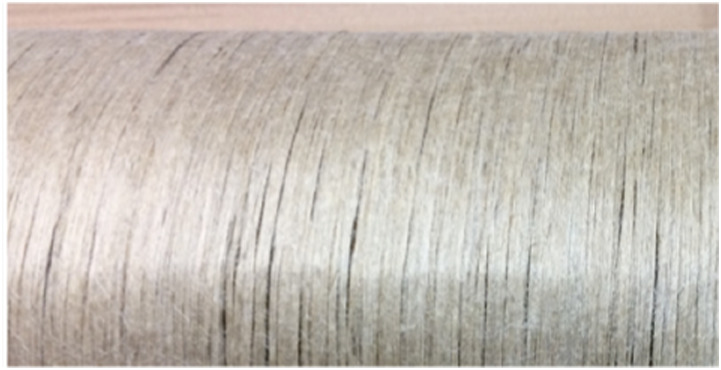
Bounded flax reinforcement fabric from Texonic

In addition to the loose flax fibre composite material, we manufactured a material using a newly developed flax reinforcement fabric from Texonic,^29^ see Figure 4. The fabric consists of unidirectional flax fibres maintained with a sprayed adhesive on one side and is meant to be used as a decorative ply in layups, in the same way as the Ekoa material by Lingrove.^30^

**Figure 5.**
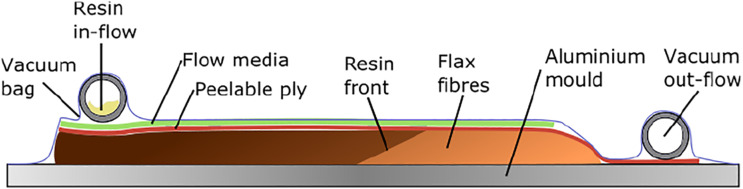
Vacuum assisted resin transfer moulding process schematics.

To obtain the required thickness for a guitar fretboard blank, a ${\left[0_{45}\right]}_{t}$ layup was manufactured using the resin infusion process. Only zero degree plies were used to get as close as possible to the $E_{T}/E_{L}$ ratio of fingerboard woods. A polished aluminium flat mould was used. Because the individual fibres would easily break apart from the reinforcement fabric, they were stacked on the mould using *InstaTack* fixative agent. The infusion was performed in the fibre direction so as to allow any gas bubbles to escape trough the channels between the individual fibres. For the second material, an infusion media was used to facilitate the resin flow across the part (see Figure 5).

For both manufacturing techniques, a blend of epoxy resin pigments was added to reproduce the colour of fingerboard woods. A combination of carbon black (5%) and ferrous oxide brown (1%) from Rayplex Limited^31^ was used to mimic the colour of Ebony wood.

**Figure 6.**
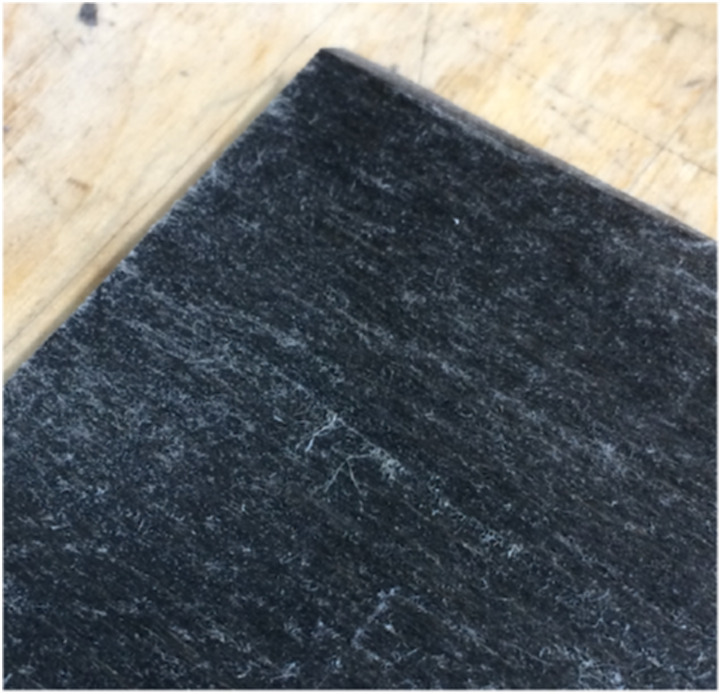
Coarsely planned flax composite material sample.

Machinability tests were performed using a wood chisel and a wood planner. The material made with adhesive bonded flax was deemed superior as a fingerboard material, because the seemingly higher fibre content made it behave more like wood when machined. The material can be machined with a wood planner to remove arbitrary thin shavings without delamination, see Figure 6.

### Physical properties of the composite material

**Figure 7.**
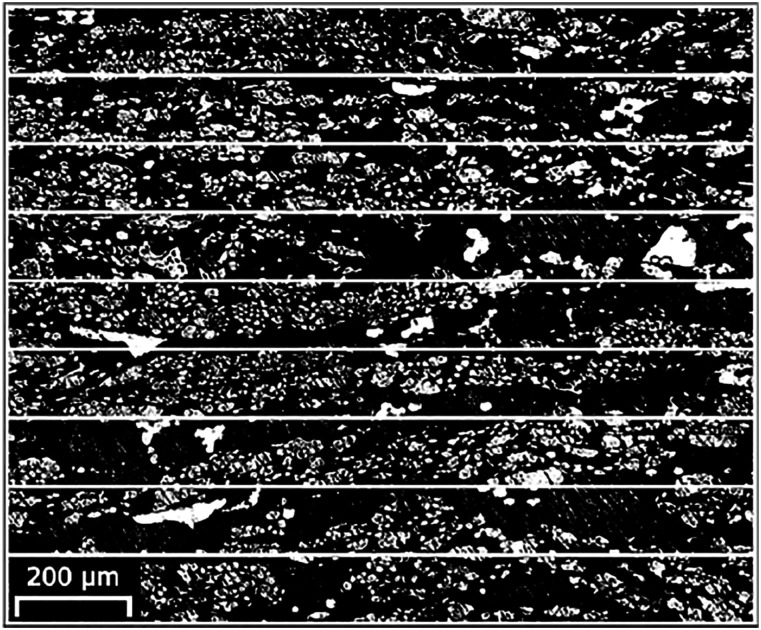
Microscopy picture with binary mask and grid to show fibre distribution.

Fibre volume fraction and porosity content of the composite material were measured. The fibre volume fraction was measured from microscopy pictures (see Figure 7) using the Fiji distribution of the ImageJ software.^32^ The microscopy pictures were stitched together and a binary mask was applied where the black content was taken as the resin and void, and the white content the fibres, resulting in a fibre volume fraction of 29%. This result can be validated by calculating the mass ratio of the dry fibres used in the parts and the final part weight which is equal to 28%.

The machinability and homogeneity trough-the-thickness of the composite material are due to the fibre distribution across the part thickness. Because of the absence of stabilizing threads in the reinforcement fabric, the flax fibre can shift when the vacuum pressure is applied. The displacement of the fibres in the thickness direction disrupts the ply sequence, thus preventing the formation of resin rich planes between plies where uncontrolled shear rupture is possible. This allows the manufacturing of a material where properties in the lateral directions are very similar to those in the thickness direction.

The porosity content was computed from the part density and fibre/matrix mass fractions with equation (1) from Monti et al.^33^

(1) $$V_{v}=1-\rho_{c}\left(\left(\frac{\phi_{p}}{\rho_{r}}\right)+\left(\frac{1-\phi_{p}}{\rho_{m}}\right)\right)$$

where *ρ*_*c*_ is the composite part density measured from its mass and volume, *ρ*_*r*_ is the fibre density taken from Ref^34^, *ρ*_*m*_ is the matrix density obtain from the manufacturer^28^ and *ϕ*_*p*_ is the fibre mass fraction obtained from the measured fibre volume fraction. The part porosity content is 6 ± 2*%*, which is in accordance with typical values found in the literature for flax composites.^33^

The bio-based content was computed from the resin and flax fabric mass fractions. The flax was taken to be 100% bio based and according to the manufacturer the Infugreen810 resin mixed with the hardener is 31% bio-based. With a fibre mass fraction of 42% computed from the measured fibre volume fraction, the material is 60% bio-based.

### Mechanical properties comparison with usual fingerboard woods

The mechanical properties of the flax composite material were measured and compared with those of ebony wood (*Diospyros Ebenum*) and rosewood (*Dalbergia latifolia*), commonly used woods in stringed instrument fingerboards.^1^ The longitudinal *E*_*L*_ and tangential *E*_*T*_ modulus were measured in accordance with the ASTM D3039 test method.^35^ The longitudinal modulus test samples were manufactured with dimensions (150 × 12.5 × 1) and the lateral modulus samples dimensions (100 × 25.4 × 3), dimensions are length, width and thickness in millimetres. The ebony wood properties were taken from Liu et al.^23^ The results are presented in Table 1.

**Table 1.** Flax composite and fingerboard woods properties.

Material	*E*_*L*_ (GPa)	*E*_*T*_ (GPa)	*ρ* (*g*/*cm*^3^)
Flax	19 (1)	3.1 (0.2)	1.18 (0.06)
Ebony	18 (2)	—	1.21 (0.01)
Rosewood	14 (1)	—	0.75 (0.01)

Standard deviations are shown in parentheses

To ensure the commercial viability of the flax composite material for an industrial scale musical instrument production, its compatibility with polyvinyl adhesive was investigated. The adhesive bond strength with polyvinyl adhesive was measured in accordance with the ASTM D5869 test method,^36^ as a reference the epoxy resin adhesive shear strength was also measured. For every lap shear test sample, two composite material samples were manufactures with dimensions (101.6 × 25.4 × 3) millimetres, the samples were bonded on a 25.4 *mm* distance, allowing for a 6.45 *cm*^2^ overlap bond surface. As recommended per the adhesive manufacturer guidelines, fixturing pressure was used by means of a manual clamping device. The shear strength of bonded joints in wood has significant variability across wood species and testing conditions, thus experimental results are not easily reproducible. According to Chahud et al.,^37^ the shear strength of bonded joints with polyvinyl adhesive in wood is between 2 and 10 MPa, the results are presented in Table 2. The adhesive bond strength using polyvinyl adhesive remains lower than the epoxy bond strength, but is in the interval of strengths for bonded wood joints.

**Table 2.** Adhesive shear strength of fingerboard materials.

Material-adhesive	Shear strength (MPa)
Flax-epoxy	6.8 (0.1)
Flax-polyvinyl	2.8 (0.4)
Wood-polyvinyl	2–10^1^

Standard deviations are shown in parentheses, 1.^37^

### Aesthetics

**Figure 8.**
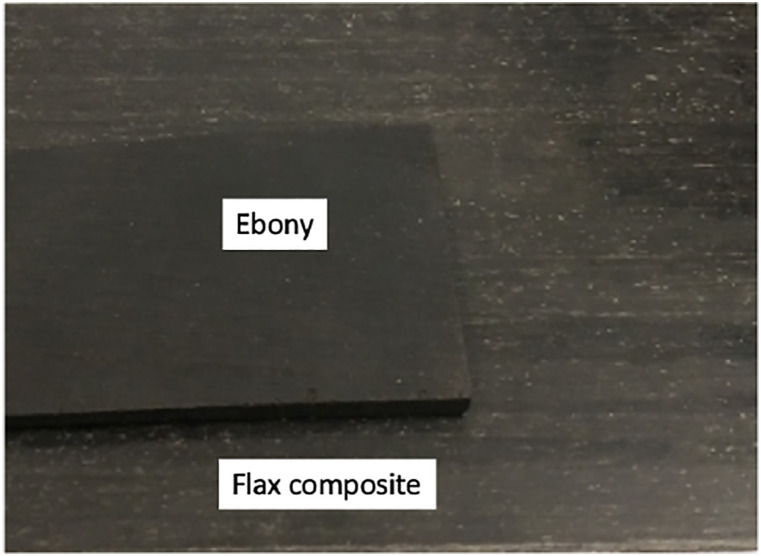
Colour comparison between finished ebony wood and flax composite material with epoxy pigments.

The blend of epoxy pigments used allowed for a close match to the colour of ebony wood, see Figure 8. As the flax fibres have a pale colour, any dark tone could be achieved by adjusting the pigment quantities, such as the colour of rosewood. According to Vincent Cléroux, a luthier at Godin Guitars, some guitar models are best made with specific colour tones of wood to match the overall look of the instrument. Sourcing a specific wood colour can be troublesome; thus, our flax based material could increase productivity and allow more possibilities in colour tones.

The fingerboard surface is normally polished to a glossy finish, for aesthetics purposes and to allows the musician’s fingers to better slide when he is playing. As the part has to be machined before the surface is finished, the surface finish cannot rely on a good mould finish and treatment, as would normally be done for composite material parts. Due to the material homogeneity, an excellent surface finish was obtainable by abrasion with 200–500 grit sandpaper and polishing with wood finish oil.

### Material industrialisation

Much of the present work was focused on the development of a material using the resin infusion process as it is easily scalable and suitable for sheet parts. The manufacturing processes for guitar fretboards currently used by instrument makers require fixed dimension rectangular blanks which can be cut from large sheets of infused flax composite material.

To assert the industrial viability of the flax composite material, the price of the raw material needed to produce a single guitar fretboard blank was calculated, as it is the instrument part with the most synthetic material alternatives to be compared to. The gross price of flax reinforcement fabric was obtained from Texonic and the resin price was obtained from a Sicomin sales representative. The price of the required raw materials to produce a single fretboard blank is 33.00 $, with the resin costing 8.50 $ and the flax reinforcement fabric costing 24.50 $ (prices are in Canadian dollars). The resin transfer moulding consumables cost and labour cost were not included in the price, as they would differ greatly from the actual costs of producing the material on a large scale.

The preliminary cost assessment demonstrates that the flax composite material could be viable on an artisan scale, meaning that it could be competitive to the Rocklite fretboard material currently used by luthiers as a synthetic alternative to exotic woods. However, the cost of the raw materials should be reduced for it to be viable on an industrial scale, as it is still significantly higher than the retail cost of materials used in large scale production (rosewood, ebony and Richlite).

For the sake of comparison, customer retail prices were used, the prices were taken from the wood to works Web site, a popular retail Web site used by luthiers,^38^ the prices are shown in Table 3.

**Table 3.** Guitar fretboard material prices comparison.

Material	Price (CAD $)
Rosewood	14.00
Ebony wood	27.00
Richlite	24.00
Rocklite	45.00
Flax composite	33.00^a^

^a^Price of raw materials only.

## Guitar Prototype

In order to demonstrate the viability of our fingerboard material, we built a prototype by replacing the fretboard on a used guitar. A generic half scale classical guitar was used.

**Figure 9.**
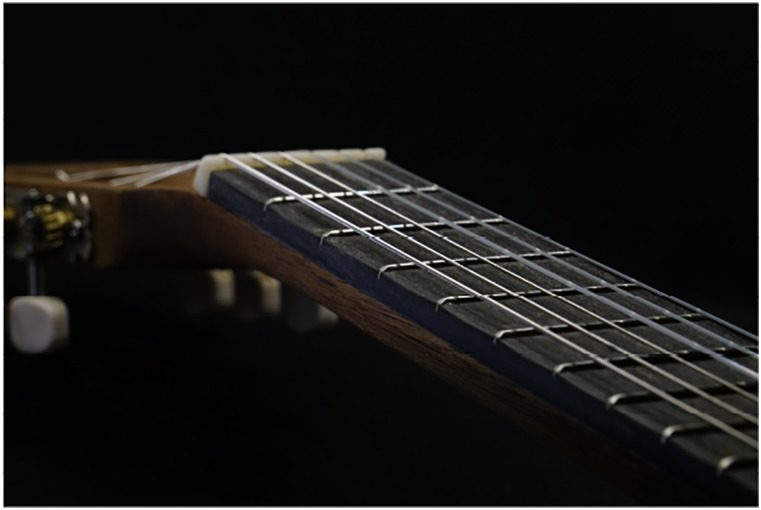
Guitar fretboard prototype.

The finished flax fretboard on the guitar prototype is shown at Figure 9. A sound sample of the guitar prototype with a flax fretboard being played can be found here.^39^

### The guitar prototype assembly

To assemble the prototype, first the old fretboard was removed from the guitar by heating the adhesive and loosening the joint between the two parts. Then, following the guitar building advice and techniques from Gore and Gilet,^20^ the flax fretboard blank was squared with a manual wood planner, allowing the fret slots to be cut precisely perpendicular to the fretboard. To ensure the note played were accurate to the guitar scale (the distance between the bridge and the top, see Figure 1) the old fretboard fret placement was used as a reference.

A manual saw was used to machine the fretboard slots, then the fretboard blank was roughly cut to the neck dimensions. To bond the fretboard onto the neck, we used epoxy adhesive, as it allowed to fill the imperfections between the fretboard and neck surface. If the two surfaces had been perfectly planar, polyvinyl adhesive would have been used; thus, its suitability for fretboard assembly is yet to be completely demonstrated. To securely bond the fretboard to the neck it was clamped with numerous clamps and a metal bar acting as a stabiliser, as recommended by Gore and Gilet .^20^ After the fretboard was bonded to the neck, it was trimmed to neck dimensions using a precision hand saw and a sanding block, the guitar assembly was then ready for the frets to be inserted.

### Fret installation

The guitar normally has frets which complicates the building process, as they have to be pressed into the fretboard material. This allowed us to test the flax composite material compatibility with fret inserts, demonstrating its viability for other fretted instruments (banjo, ukulele and others).

According to Gore and Gilet^20^ the frets can either be pressed in before or after the fretboard is bonded onto the guitar, we chose to press them after, as the guitar neck was already finished this allowed us to precisely sand the fretboard to the neck dimensions. The machined fret slots had a width of 0.80 mm and a depth of 2.5 mm, a guide was used to ensure constant depth and perpendicularity when cutting the slots. To install the frets, a polyvinyl adhesive was deposited in the slots and the frets were hammered in. The fret slot width used was tested for wood and for the flax fretboard, the frets could be inserted without trouble or excessive bending of the neck while being securely in place for both, an increase of the width used for wood was not necessary as opposed to carbon fibre fretboards.^40^

### Surface finish and playability

The fingerboard (and neck) are the main parts of the instruments with which the musician interacts. Notwithstanding the instruments aesthetics and acoustics, how the musician feels the instrument is of primordial importance.

To obtain a good surface finish, the flax fretboard was sanded using sanding paper from 200 to 500 grit, and then the surface was polished using a cotton towel and wood polishing oil recommended by Lutherie Denalt.^8^ A good surface finish was harder to obtain using the flax material as small strands of fibre would detach from the resin matrix when sanding with coarser paper, these small strands would then have to be sanded out using the smaller grit paper. Also, the matrix hardness which is higher than common wood hardness, made removing large amount of matter harder, but allows for a more durable material in the long run.

The surface finish of the flax fretboard and its perceived hardness was deemed acceptable by Lutherie Denalt^8^ and the finished guitar prototype demonstrated the possible viability of the flax fibre material as a fretboard material according to Godin Guitars luthier Vincent Cléroux.^41^

## Conclusion

To conclude, a suitable flax reinforced composite material fingerboard material was developed by doing iterations on the manufacturing method using different types of flax reinforcement. The approach where a non-laminate and homogeneous trough-the-thickness material was produced allowed for good machinability using common lutherie tools, thus demonstrating compatibility with existing production equipment. The material manufactured with a novel type of unidirectional flax reinforcement fabric stabilised with adhesive instead of knitting threads was chosen due to its wood-like behaviour when machined. The physical and mechanical properties of the flax material were measured and were found to be similar to those of rosewood and ebony, which are commonly used fingerboard materials. To demonstrate the material viability as a fretboard material, a guitar prototype was manufactured by replacing the wood fretboard of a classical guitar. Guitar frets were installed into the flax material, which was shaped into a guitar fretboard and a good quality finish was obtained. The resulting prototype was evaluated by experienced luthiers. The material viability on an industrial musical instrument production line remains to be proven, as well as its economical viability, but it is at the time of writing this article the synthetic fingerboard alternative having the highest bio-based content with a mass fraction of 60% bio-based materials.
